# A Comparative Study on Clinical Efficacy of Clobetasol and Betamethasone in Orabase in Combination With Clotrimazole in the Management of Oral Lichen Planus

**DOI:** 10.7759/cureus.99977

**Published:** 2025-12-23

**Authors:** Pratima Soni, Komali Garlapati, Divya Harika Pedada, Mahitha Konda, Anuja Kammari, Srushti Bachuwar

**Affiliations:** 1 Oral Medicine and Radiology, Panineeya Institute of Dental Sciences and Research Centre, Hyderabad, IND; 2 Oral Medicine and Radiology, Sri Balaji Dental College, Hyderabad, IND

**Keywords:** betamethasone, clobetasol, corticosteroids, oral lichen planus, randomized trial

## Abstract

Context: Oral lichen planus (OLP) is a chronic inflammatory mucocutaneous disease characterized by pain and burning sensation. As etiology remains unclear, the treatment of OLP is focused mainly on reducing symptoms through modulation of local immune response.

Aims: The aim of this study is to evaluate and compare the clinical efficacy of Clobetasol 0.05% orabase and Betamethasone 0.05% orabase, both combined with Clotrimazole 1%, in managing symptomatic OLP.

Methodology: A double-blind, randomized clinical trial was conducted on 30 patients diagnosed with OLP at a tertiary dental hospital. Patients were randomly divided into two groups: Group A (Clobetasol + Clotrimazole) and Group B (Betamethasone + Clotrimazole). Clinical response was assessed at two and four weeks using the Global Assessment Scale for lesion size and the visual analogue scale (VAS) for burning sensation. The collected data were analyzed using IBM SPSS Statistics for Windows, Version 17 (Released 2009; IBM Corp., Armonk, New York, United States).

Results: At the end of four weeks, the change in mean size obtained was score 2.0±0.95 in Group A and 1.66±1.4 in Group B (p-value = 0.51), indicating that both the study drugs had a similar decrease in the global assessment of efficacy scores with no significant statistical difference. Mean change in the VAS score at the end of four weeks revealed a decrease in the mean score of 4.66±2.30 in Group A and a decrease by 4.00±1.80 score in Group B (p-value=0.44), indicating no statistical significance.

Conclusions: Both clobetasol and betamethasone demonstrated comparable efficacy in reducing pain and lesion size among patients with OLP. This finding suggests that either regimen can serve as a viable therapeutic option in the management of OLP. Considering their similar clinical outcomes, the choice between these corticosteroids may depend on factors such as drug availability, patient tolerance, cost, and physician preference.

## Introduction

Lichen planus is a chronic inflammatory disease affecting the mucocutaneous tissues in about 0.5% to 2% of the general population [1]. It includes reticular, atrophic, erosive-ulcerative, bullous, and pigmentous forms [2-4]. Treatment of oral lichen planus (OLP) includes both pharmacological methods, such as corticosteroids, calcineurin inhibitors (cyclosporine, tacrolimus, and pimecrolimus, retinoids, dapsone, mycophenolate mofetil, etc., and non-pharmacological methods like PUVA (Psoralen and Ultraviolet A) therapy, photodynamic therapy and laser therapy [4]. As no current therapeutic options offer a definitive cure for OLP, the primary goal of treatment is to provide symptomatic relief by modulating the local immune response. Corticosteroids are commonly used and often provide significant relief, either as monotherapy or in combination with other immunomodulatory agents. Their efficacy is attributed to their ability to modulate both inflammatory and immune responses by stabilizing lysosomal membranes and reducing lymphocytic infiltration [5].

Corticosteroids, especially topical formulations, remain the mainstay of treatment in OLP. Topical corticosteroids, such as triamcinolone acetonide, betamethasone phosphate, fluocinonide acetonide, and clobetasol propionate, are commonly employed in the management of OLP due to their anti-inflammatory and immunosuppressive properties. However, their efficacies vary depending on several factors, including their inherent potency, formulation (ointment, gel, paste), degree of mucosal penetration, and the extent and severity of the lesions [6]. 

The use of topical corticosteroids in orabase has been shown to improve the clinical efficacy of these medications and also, in combination with antifungal agents, was found to control coexisting candidiasis in OLP (mainly in erosive and atrophic forms) and prevent corticosteroid-induced candidiasis. Very few studies have been conducted on topical clobetasol and betamethasone in OLP and no comparison has been reported in the literature between topical clobetasol and betamethasone in the treatment of OLP. This study was designed to evaluate and compare the clinical efficacy of topical Clobetasol 0.05% orabase and Betamethasone 0.05% orabase, both in combination with Clotrimazole 1%, in the management of symptomatic OLP, as assessed by changes in lesion severity. The secondary objectives included evaluation of pain relief and monitoring for adverse effects.

## Materials and methods

Study design and participants

**Figure 1:**
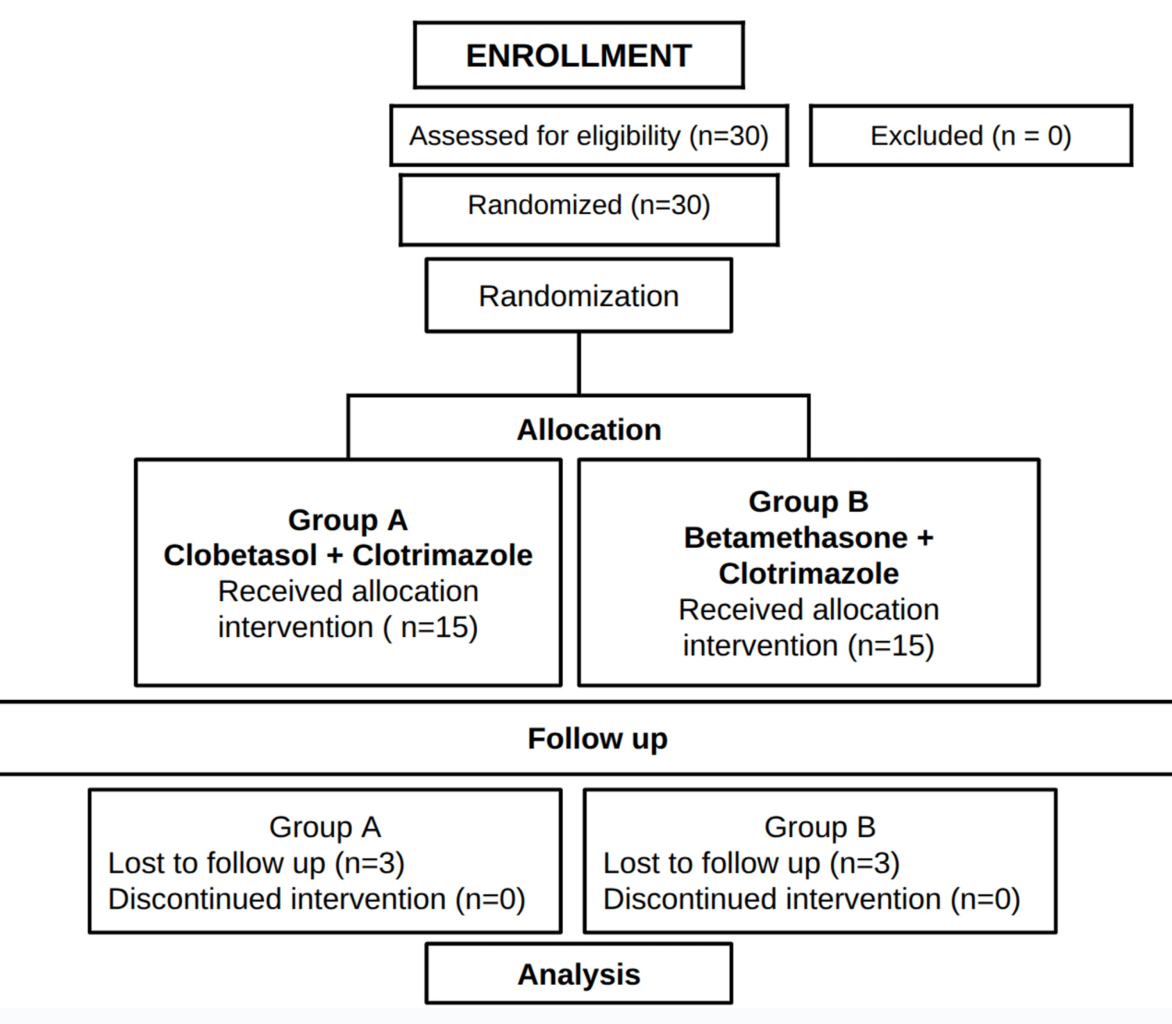
CONSORT flowchart. CONSORT: Consolidated Standards of Reporting Trials The figure is the author's creation.

A double-blind, randomized clinical trial was conducted at Panineeya Institute of Dental Sciences and Research Centre, Hyderabad, India, between November 2015 and March 2017, with a four-week treatment duration. The study involves 30 patients with confirmed OLP who were randomly allocated into two groups: Group A (15 patients who received Clobetasol 0.05% in orabase with Clotrimazole 1%) and Group B (15 patients who received Betamethasone 0.05% in orabase with Clotrimazole 1%). The Institutional Ethical Review Board approved the study (PMVIDS&RC/IEC/OMR/DN/0003-15). The clinical trial was prospectively registered at ClinicalTrials.gov (Identifier: NCT03026478) with the protocol ID PMVIDS&RC/IEC/OMR/DN/0003-15. It adhered to the ethical guidelines set forth by the World Medical Association's Declaration of Helsinki. All participants gave their consent, and confidentiality was upheld throughout the study. There was no patient or public involvement in the design, conduct, or reporting of this research. The reporting of this study follows the CONSORT (Consolidated Standards of Reporting Trials) guidelines (Figure 1).

Eligibility criteria 

The study enrolled healthy patients aged 18 years or older who presented with a definitive clinical and histopathological diagnosis of OLP. To be eligible for inclusion, participants were required to have symptomatic erosive or atrophic lesions, characterized by a self-reported pain or burning sensation. Patients were excluded from the study if they presented with any other concurrent mucosal or cutaneous diseases, mucocutaneous lichen planus, or lichenoid reactions (whether drug-induced or contact-related). Individuals reporting deleterious habits, such as smoking or gutka chewing, were not included. Additionally, the study excluded pregnant or lactating women, patients with systemic diseases where corticosteroid use is contraindicated, and those with a known allergy or hypersensitivity to clobetasol, betamethasone, or clotrimazole.

Sample size calculation 

The sample size was determined based on an a priori power analysis. We anticipated a robust response to the intervention and aimed to detect a clinically meaningful difference of 1.1 units in the Thongprasom clinical score between groups. Assuming a standard deviation (𝜎) of 1.0 and setting the power (1- 𝛽) at 80% with a two-sided significance level (⍺) of 5%, the required sample size was calculated to be approximately 14 patients per group. To account for potential attrition, we targeted a total enrollment of 30 patients (15 per group).

Randomization and allocation concealment

The random allocation sequence was generated by a coordinator, who was not involved in patient recruitment or assessment, using a computer-generated randomization sequence with a 1:1 allocation ratio (equal numbers in Group A and Group B). Allocation concealment was maintained using a centralized third-party allocation process using sequentially numbered, opaque, sealed envelopes (SNOSE). An independent coordinator, who was not involved in the recruitment or assessment of participants, safeguarded the randomization sequence. Assignment was revealed only after enrollment to prevent selection bias.

Blinding

**Figure 2:**
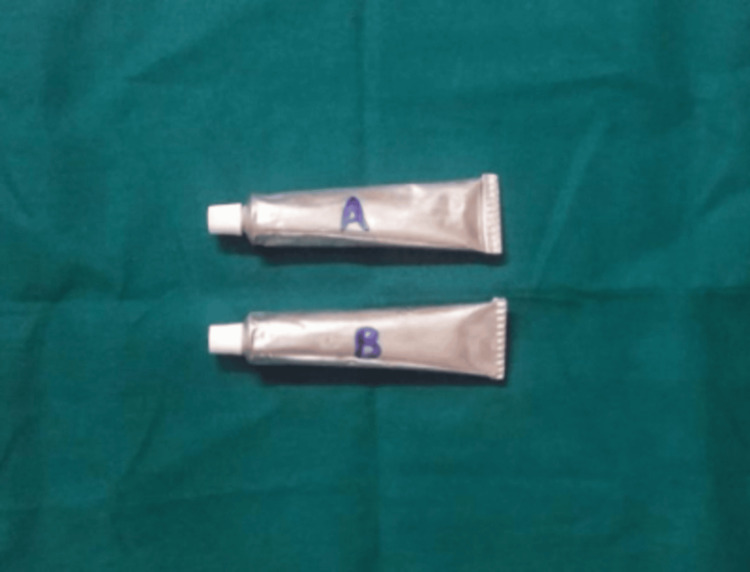
Two topical medicaments prepared in orabase: Tube A (Clobetasol + Clotrimazole) and Tube B (Betamethasone + Clotrimazole), labeled for blinding in the clinical trial.

This was a double-blind trial. Participants and the outcome assessors (the investigators who evaluated the Thongprasom and VAS scores) were blinded to the treatment assignments. Blinding was achieved by preparing the two medicated orabase pastes (Group A: Clobetasol + Clotrimazole; Group B: Betamethasone + Clotrimazole) to be identical in appearance, color, consistency, and smell. The tubes were labeled only with the codes 'A' and 'B' (Figure 2).

The code key was held securely by the unblinded coordinator until the final data analysis was complete. The personnel who prepared the study medications were not involved in patient care, assessment, or data analysis.

Intervention

The 0.05% topical Clobetasol propionate gel and 0.05% betamethasone were prepared by Hyderabad Karnataka Educational Society’s Institute of Pharmacy, Karnataka. Preparation involved dissolving clobetasol propionate raw powder in water with continuous stirring, adding carbopol 934p, allowing the mixture to settle for 36 hours, and then incorporating TEA (triethanolamine), M & P (methyl and propyl) parabens, ethanol, and finally sterilizing by autoclaving. Preparation of topical betamethasone dipropionate gel followed a similar process with specific measurements for each component and sterilization by autoclaving.

Patients were asked to apply the medication after meals, twice daily, as a thin layer over the lesion using a clean and dry finger. After one hour of application of study medication, patients were asked to apply clotrimazole 1% and abstain from eating or drinking anything for one hour post-application. Patients were prohibited from using any other emollient during the application of the study medication. If allergic manifestations were evident during the application of the medication, patients were asked to discontinue the medication and report to the hospital immediately.

Clinical assessment 

Clinically, the erosive and atrophic lesions were included and evaluated using the clinical criteria scale developed by Thongprasom [7]. Burning sensation was assessed using the Visual Analogue Scale (VAS) [8], patients were asked to score their intensity of burning with scores ranging from 0 (no pain) to 10 (extreme pain). Treatment response was evaluated at the end of two and four weeks using the Thongprasom clinical criteria [7].

Harms and interim analysis 

Adverse events (AEs) were monitored and were told to inform in case of any unwanted side effects, signs of local irritation, or systemic symptoms and were graded into mild, moderate and severe according to their severity. No interim analysis was performed due to the short duration of the study.

Statistical analysis

Data was compiled in a standardized format, and statistical analysis was performed using IBM SPSS Statistics for Windows, Version 17 (Released 2009; IBM Corp., Armonk, New York, United States). The demographic data and baseline characteristics were summarized using descriptive statistics. The primary analysis was conducted on the per-protocol population of the 24 patients who completed the full four-week follow-up and adhered to the treatment schedule. Independent sample T-tests were used to compare the mean change from baseline in Thongprasom clinical criteria score [7] and VAS scores [8] between Group A and Group B at the two-week and four-week follow-up endpoints. Paired sample t-tests were used to compare the mean lesion size and VAS scores at two and four weeks against the baseline value within each individual treatment group. A two-sided p-value of less than 0.05 was considered statistically significant.

## Results

Age and gender distribution

Patients ranged from 30 to 65 years old. Group A had a mean age of 48.83 years (SD = 8.72), and Group B had a mean age of 48.33 years (SD = 8.35). There were 19 female patients and five male patients overall. In Group A, 83.3% were female patients and 16.7% were male patients. In Group B, 75% were female patients and 25% were male patients.

Clinical types of lichen planus 

The study included patients with erosive and atrophic lichen planus. In Group A, 50% had erosive, and 50% had atrophic lichen planus. In Group B, 66.7% had erosive, and 33.3% had atrophic lichen planus (Table 1).

**Table 1:** Distribution of clinical forms (erosive and atrophic) of oral lichen planus in clobetasol and betamethasone groups.

Groups	Clinical types	Frequency	Percent
Group – A Clobetasol	Erosive	6	50
	Atrophic	6	50
	Total	12	100
Group – B Betamethasone	Erosive	8	66.7
	Atrophic	4	33.3
	Total	12	100

Distribution of lesions by site

**Figure 3:**
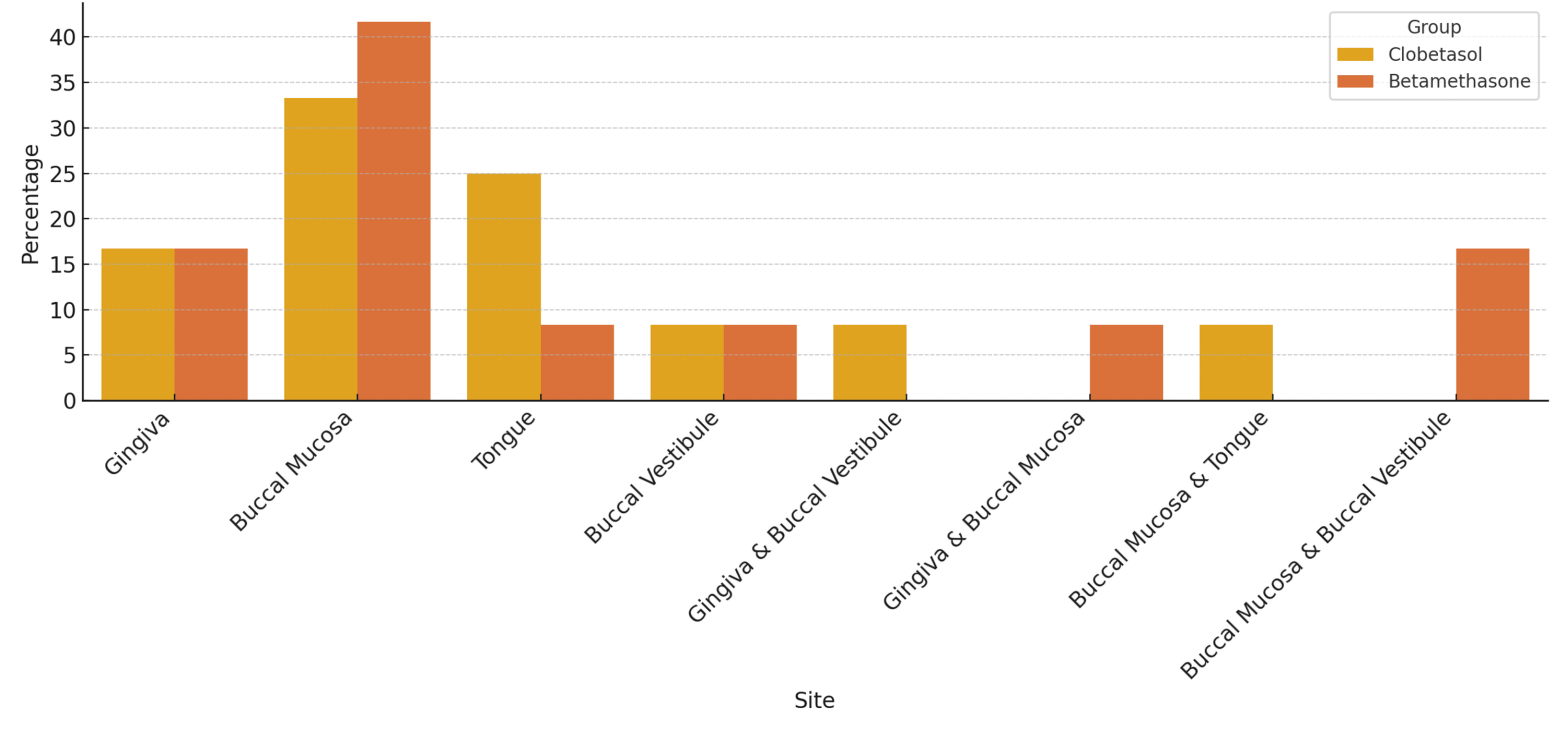
Site-wise distribution of lesions in patients with oral lichen planus, showing percentages affected in each site for both treatment groups.

In Group A, lesions were primarily on the buccal mucosa (33.3%), followed by the tongue (25%), gingiva (16.7%), buccal vestibule (8.3%), gingiva and buccal vestibule (8.3%), and buccal mucosa and tongue (8.3%). In Group B, lesions were mostly on the buccal mucosa (41.7%), followed by the gingiva (16.7%), buccal mucosa and buccal vestibule (16.7%), tongue (8.3%), buccal vestibule (8.3%), and gingiva and buccal mucosa (8.3%) (Figure 3).

Lesions were initially scored using Thongprasom criteria and treatment response was assessed every week until four weeks using the Global Assessment Scale.

Comparison of change in mean size of the lesion within group A and group B at 4 weeks

**Figure 4:**
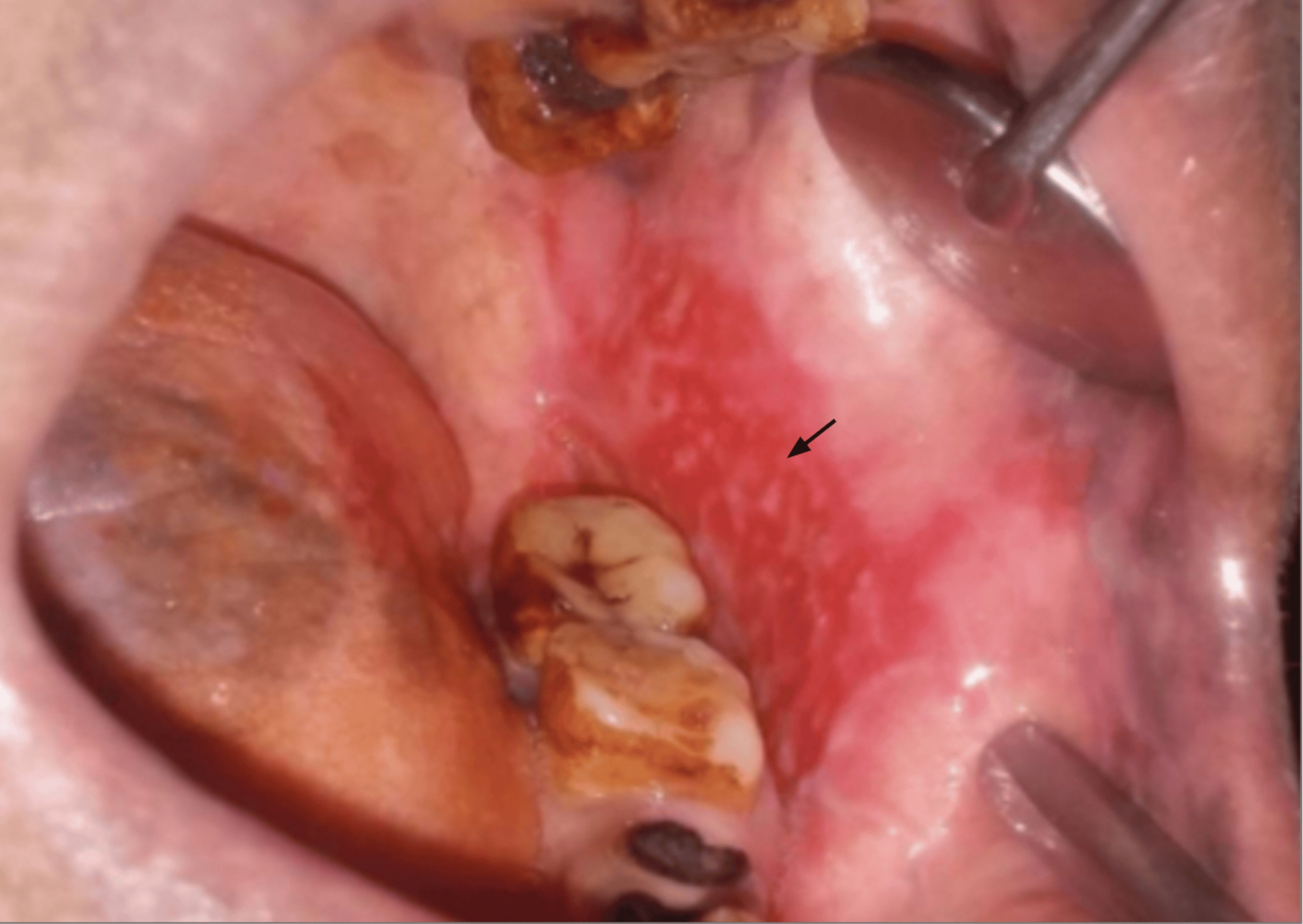
Oral lichen planus on the left buccal mucosa in a Group A patient before treatment with Clobetasol 0.05% in orabase. Arrow indicates the lesion margin

**Figure 5:**
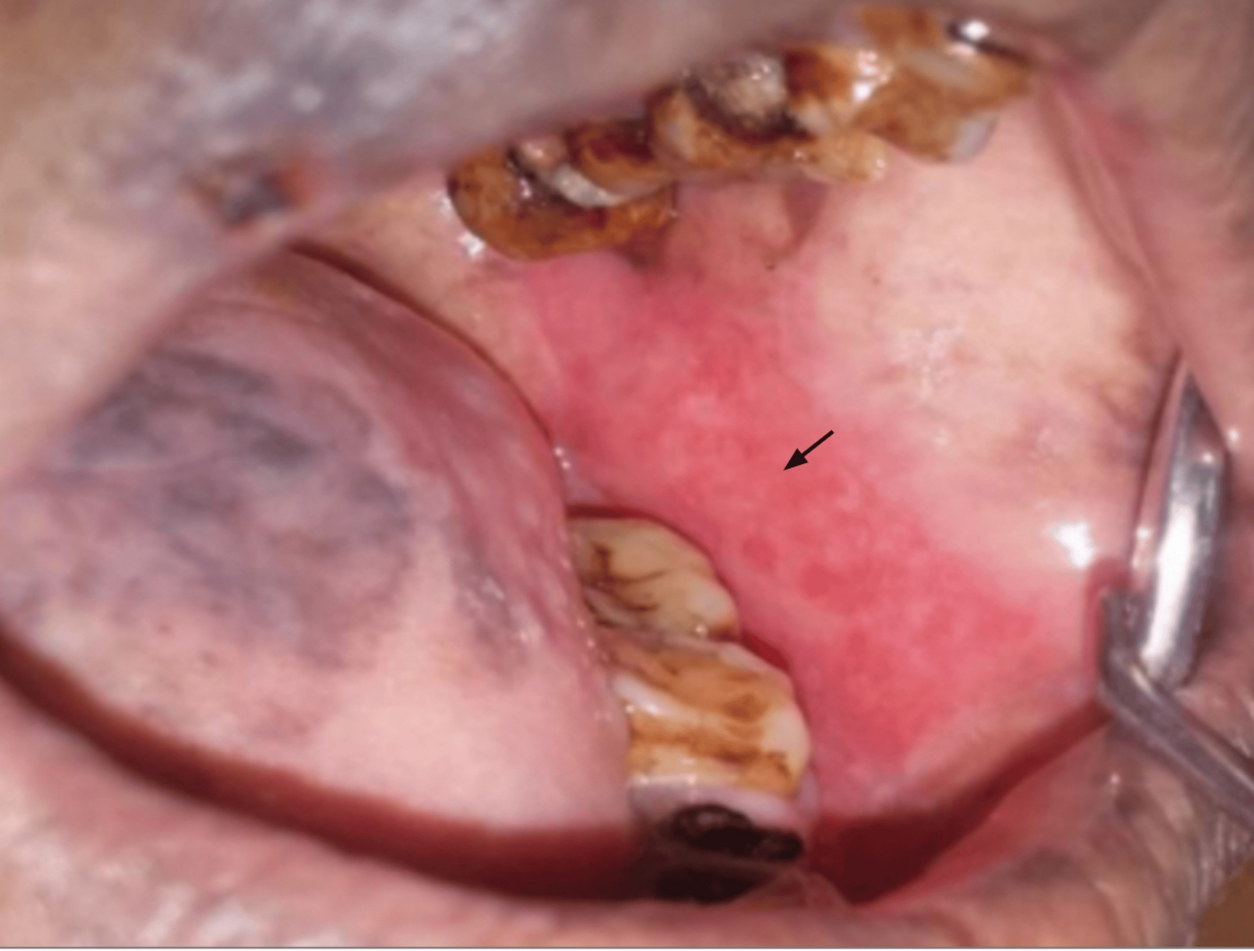
Post-treatment resolution of the oral lichen planus lesion on the buccal mucosa in a Group A patient after four weeks of therapy with Clobetasol 0.05% in orabase. Arrow indicates the reduction of the lesion

**Figure 6:**
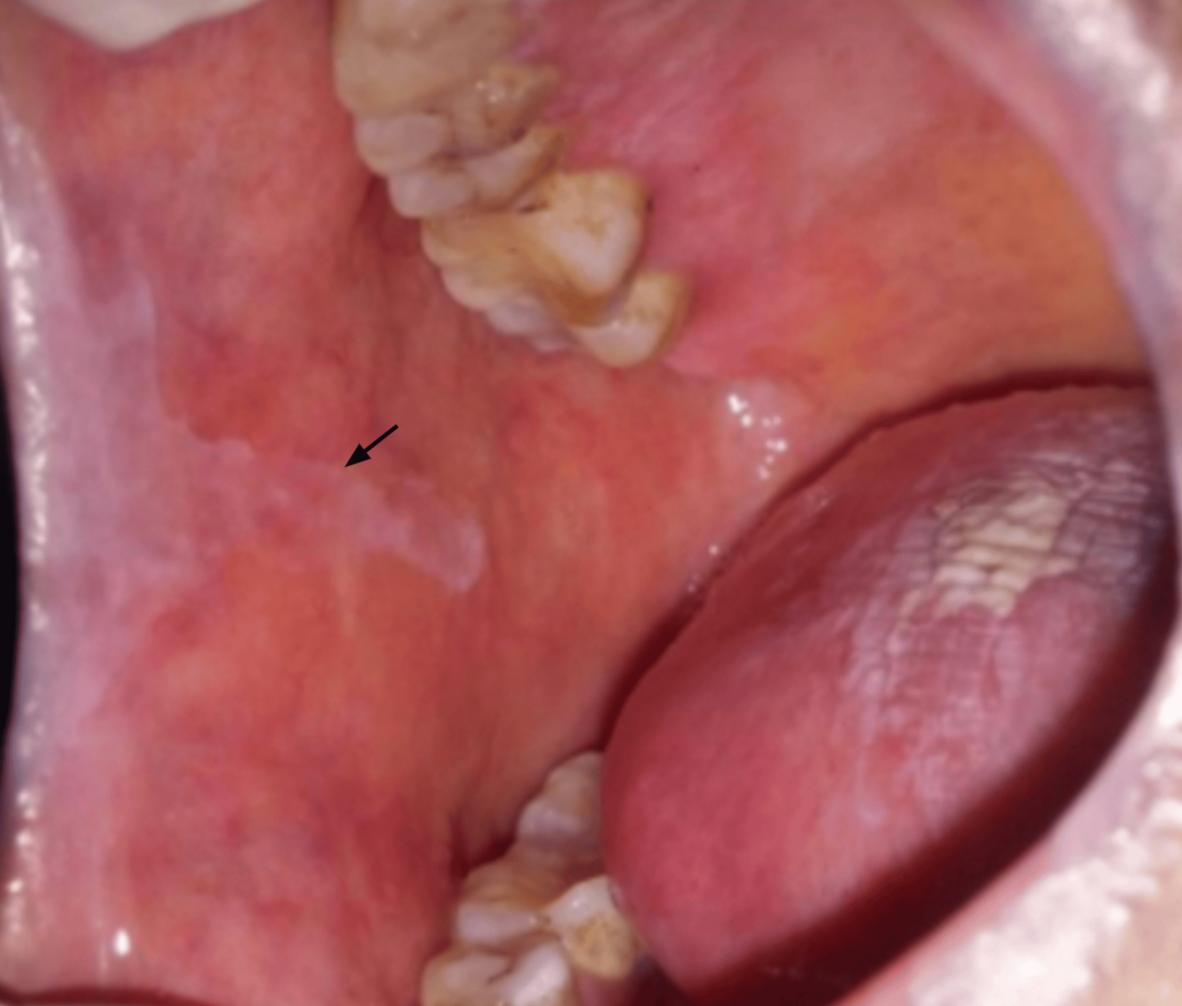
Oral lichen planus lesion on the buccal mucosa in a Group B patient before treatment with Betamethasone 0.05% in orabase. Arrow indicates the lesion margin

**Figure 7:**
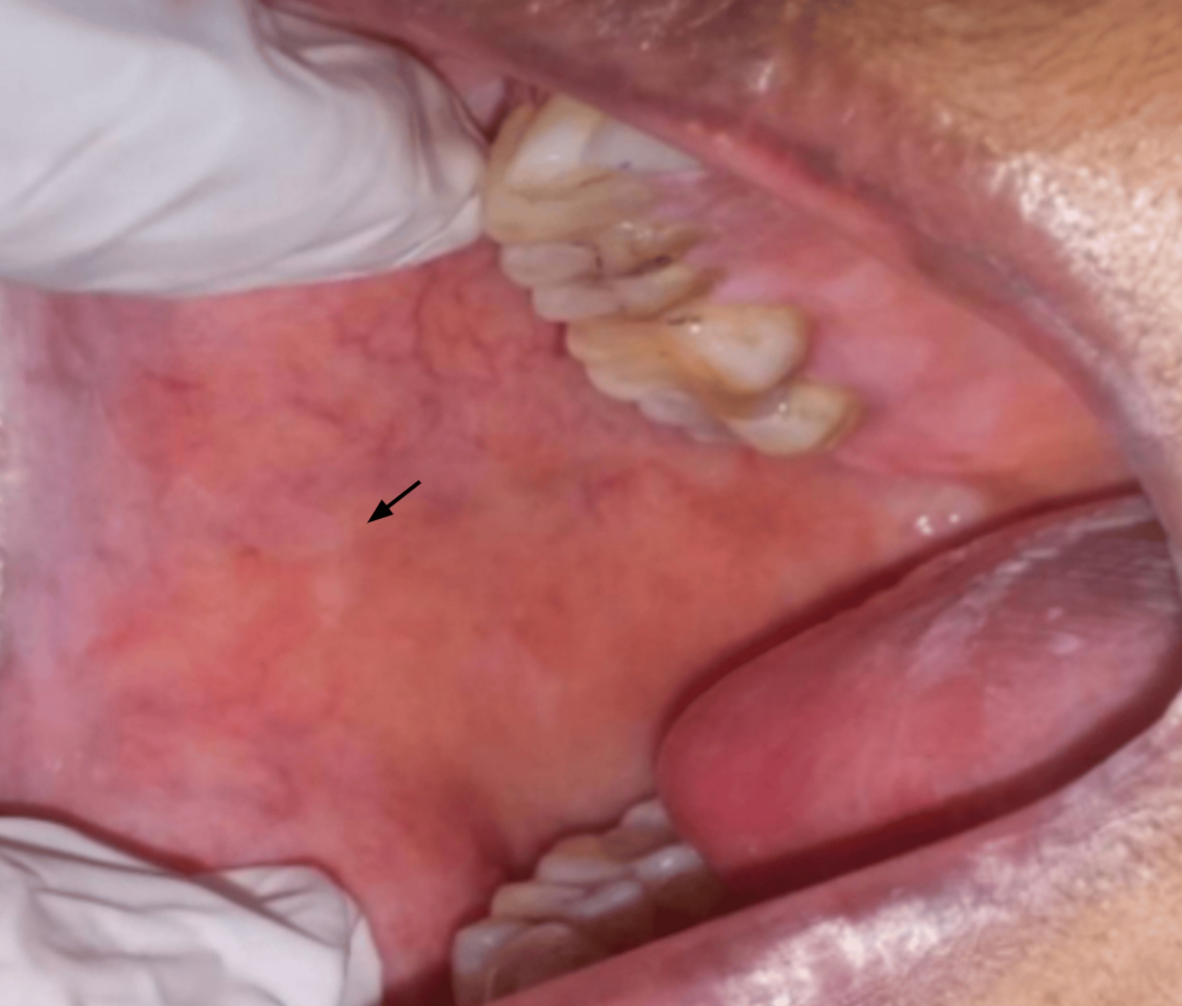
Clinical improvement in a Group B patient showing reduction of the oral lichen planus lesion on the buccal mucosa after four weeks of treatment with Betamethasone 0.05% in orabase. Arrow indicates the reduction of the lesion.

Within-group analysis for Group A showed a highly significant reduction in lesion size from baseline (Figure 4) to the four-week endpoint (Figure 5), with a mean score change of 2.0+/- 0.95 (t = 8.16, df = 11, p < 0.0001). Similarly, Group B exhibited a meaningful reduction in lesion size relative to pre- treatment level (Figure 6). By the end of four weeks, treatment with Betamethasone 0.05% in orabase resulted in a mean size reduction of 1.66 +/- 1.4 (t = 4.03, df = 11, p = 0.002), as evidenced by the post-treatment resolution shown in Figure 7.

Comparison of change in the mean score of lesion size between groups after two weeks

An independent sample t-test compared the mean change in Global Assessment of Efficacy scores after two weeks. Group A had a mean and standard deviation of 2.75±1.06 and Group B had a mean score of 2.92±1.38, and a p-value of 0.28. This indicates no significant difference between the groups in reducing lesion size (Table 2).

**Table 2:** Comparison of lesion size changes between clobetasol and betamethasone groups after two weeks, assessed using the Global Assessment Scale. Independent sample T-test; p≤0.05 considered statistically significant; T = t-statistic from Student’s t-test.

Group	Number	Mean	Standard deviation	Standard error mean	Mean difference	95% Confidence interval		T	p
Clobetasol	12	2.75	1.06	0.30	0.17	1.21	0.87	0.33	0.28
Betamethasone	12	2.92	1.38	0.40					

Comparison of change in the mean score of lesion size between groups after four weeks

An independent sample T-test compared the mean change in Global Assessment of Efficacy scores after four weeks. Group A had a mean and standard deviation of 2.0±0.95 and Group B had a mean score of 1.67±1.44, and a p-value of 0.51. This indicates no significant difference between the groups in reducing lesion size (Table 3).

**Table 3:** Comparison of lesion size change between clobetasol and betamethasone groups after four weeks, assessed using the Global Assessment Scale. Independent sample T-test; p≤0.05 considered statistically significant; T = t-statistic from Student’s t-test.

Group	Number	Mean	Standard deviation	Standard error mean	Mean difference	95% Confidence interval	T	p
Group A Clobetasol	12	2.00	0.95	0.28	0.33	0.70	0.67	0.51
Group B Betamethasone	12	1.67	1.44	0.41				

Comparison of the VAS score within group A and group B at four weeks

Comparison of VAS scores from baseline to four weeks within Group A (Figure 4, Figure 5) is statistically significant at four weeks, with t = 6.52, df = 11, p-value = 0.00003, and Group B (Figure 6, Figure 7), t = 7.14, df = 11, p-value = 0.00002, indicating a significant reduction in VAS scores over the four weeks.

Mean comparison of change in the VAS score between groups at the end of two weeks

An independent sample T-test compared the mean and standard deviation in VAS scores between the clobetasol and betamethasone groups after two weeks. The clobetasol group showed a mean decrease of 2.25±1.36, while the betamethasone group showed a mean decrease of 2.83±1.59. No significant statistical difference was found (p = 0.34), indicating similar decreases in VAS scores for both groups (Table 4).

**Table 4:** Change in visual analogue scale (VAS) scores between groups at the two-week follow-up. Independent sample T-test; p≤0.05 considered statistically significant; T = t-statistic from Student’s t-test

Group	Number	Mean	Standard deviation	Standard error mean	Mean difference	T-value	p-value
Clobetasol	12	2.25	1.36	0.39	0.58	0.97	0.34
Betamethasone	12	2.83	1.59	0.46			

Mean comparison of change in the VAS score between groups at the end of four weeks 

An independent sample T-test compared the mean change in VAS scores between two study groups over four weeks. The clobetasol group showed a mean decrease of 4.67±2.31, while the betamethasone group showed a mean decrease of 4.00±1.81, with no statistically significant difference (p = 0.44) (Table 5).

**Table 5:** Change in visual analogue scale (VAS) scores between groups at the four-week follow-up. Independent Sample T-test; p≤0.05 considered statistically significant; T = t-statistic from Student’s t-test.

Group	Number	Mean	Standard Deviation	Standard error mean	Mean Difference	T-value	p-value
Clobetasol	12	4.67	2.31	0.67	0.67	0.79	0.44
Betamethasone	12	4.00	1.81	0.52			

Clinical mean response grading after two weeks was 2.25 for clobetasol and 2.83 for betamethasone, and after four weeks, it was 4.66 and 4.00, respectively, indicating a better response for betamethasone at two weeks and for clobetasol at four weeks. Mean VAS scores were 3.83 for clobetasol and 4.08 for betamethasone, reducing to 1.08 and 1.16 at two weeks, and 1.83 and 2.41 at four weeks. Despite these differences, no significant difference was observed, indicating similar decreases in VAS scores for both groups after four weeks.

## Discussion

OLP typically affects middle-aged and elderly individuals, most commonly between the third and sixth decades of life [9-12]. In the present study, the included patient population was within this expected age range. The present study findings demonstrate a marked female predominance, which aligns with few studies [12,13]. However, some studies [3,14] reported an equal sex distribution, while others [9,15,16] reported a higher male-to-female ratio.

The different clinical manifestations of OLP can be explained based on the extent of subepithelial inflammation. Mild inflammation may provoke the epithelium to produce hyperkeratosis, whereas more intense inflammation will cause partial or complete deterioration of the epithelium, which is histologically seen as erosion, atrophy, or ulceration [17]. In the present study, patients with only erosive and atrophic types of lichen planus were included based on the Thongprasom criteria [7]. A total of 12 patients in each group were evaluated, where in Group A, 50% were erosive type and 50% were atrophic type, whereas in Group B, 66.70% were of erosive type and 33.30% were of atrophic type.

The buccal mucosa remains the most commonly affected site, consistent with findings from both global and regional studies [18]. The present study also found the buccal mucosa to be the most commonly affected site, with variations in the distribution of lesions among different patient groups. Maybe the frequency of OLP site involvement varies geographically.

Since OLP has no permanent cure, treatment focuses on symptom management, ranging from mild cases with no noticeable lesions to severe, painful cases that impair eating and quality of life. The main goals of OLP therapy are to resolve oral lesions, alleviate pain, and reduce the risk of oral cancer. Treatments include drug therapy, laser, PUVA, and surgery, with novel drug therapies being the most common. Topical drugs like corticosteroids, retinoids, immunosuppressives, immunomodulators, and systemic drugs like hydroxychloroquine and metronidazole are used. Systemic and topical corticosteroids are particularly effective [1]. In the present study, Group A received Clobetasol 0.05% and Clotrimazole 1%, while Group B received Betamethasone 0.05% and Clotrimazole 1%.

Few conducted studies on topical clobetasol propionate in a concentration of 0.05% for the treatment of symptomatic OLP [19-22]. While early research [23] focused on topical betamethasone dipropionate 0.05%, subsequent studies have evaluated a broader range of formulations and delivery methods. For instance, other studies [24-26] used buccal mucoadhesive properties of microparticles/discs of betamethasone disodium phosphate and intralesional betamethasone in the treatment of symptomatic OLP. More recent trials [27,28] compared betamethasone oral rinse, oral pastes to alternatives like NAVS naphthalan, rapamycin in treating symptomatic OLP.

One challenge in treating OLP is the development of oral candidiasis, which shares symptoms with OLP and is a common side effect of corticosteroid therapy. Prolonged corticosteroid use can damage mucosal barriers and induce local immunosuppression, increasing the risk of oropharyngeal candidiasis. To mitigate this, topical antifungal drugs, such as clotrimazole, are recommended. Clotrimazole, effective against yeasts and fungi, is widely used for oral candidiasis and has minimal systemic absorption. While generally safe, it can cause hypersensitivity and local irritation [29]. A study [30] found the incidence of oral fungal infections in OLP patients treated with corticosteroids, with no significant difference between those using prophylactic antifungals and those not. In the present study, 1% clotrimazole was used as a prophylactic antifungal, and no secondary candidiasis occurred, but four patients reported a burning sensation from clotrimazole, contrasting with Sholapurkar's study [31], which reported no effects.

The oral mucosa is constantly hydrated by saliva, producing about 0.5 to 2 liters daily. This hydration necessitates the use of hydrophilic polymeric matrices for oral transmucosal drug delivery [32]. Topical medications for the oral cavity require appropriate vehicles for mucosal adherence, such as zylactin, orabase, cyanoacrylate, and bioadhesive patches, with gels being preferred over creams or ointments due to better adhesion [1]. Carbopol is a useful component in gel systems for various applications, including buccal drug delivery, due to its favorable physical properties and drug release rates [33]. The present study used Carbopol orabase as the vehicle for clobetasol and betamethasone gels.

Considering the endpoint of treatment was to be after four weeks, at the mid-treatment point (two weeks), both clobetasol and betamethasone groups showed noticeable clinical improvement based on the global assessment of efficacy. While the betamethasone group exhibited a slightly better clinical response than the clobetasol group, the difference between the two groups was not statistically significant. By the end of the four-week treatment period, both groups demonstrated notable clinical improvement in the overall global assessment of efficacy. The clobetasol group exhibited a slightly better clinical response compared to the betamethasone group at this endpoint. However, the difference between the two groups was not statistically significant.

In the current study, pain reduction was assessed using the VAS at both two and four weeks. At the mid-treatment interval (two weeks), the betamethasone group showed a slightly greater reduction in pain scores compared to the clobetasol group. However, by the end of four weeks, the clobetasol group demonstrated a marginally better improvement in VAS scores. Despite these minor fluctuations in patient-reported pain relief between the two groups, the differences at both time points were not statistically significant. This suggests that both clobetasol and betamethasone provided comparable symptomatic relief over the treatment duration.

Other studies [19,20] also align with the present study. They reported efficacious outcomes with clobetasol propionate over mesalazine and tacrolimus 0.1% ointment in the treatment of OLP. The study [23] evaluating topical betamethasone dipropionate 0.05% concluded that it was superior to tretinoin in treating symptomatic OLP. It was found to be more effective in lesion control, symptom relief, and overall clinical improvement.

Within-group analysis showed a marked reduction in lesion size from baseline to four weeks in both Group A and Group B, with the difference being highly statistically significant in each case. This reflects the therapeutic effectiveness of both topical corticosteroid formulations in reducing the clinical manifestation of OLP. Similarly, a comparison of pain scores using the VAS within each group revealed a statistically significant reduction in patient-reported discomfort after four weeks of treatment. This suggests that both treatment protocols provided effective symptomatic relief over the short term. These findings support the clinical utility of both clobetasol and betamethasone in managing symptomatic OLP, particularly in terms of reducing lesion severity and improving patient comfort during the study period.

The present study findings align with those of another study [34], which also reported a significant reduction in pain scores over a five-week treatment period using Clobetasol 0.05% cream. Their results demonstrated gradual improvement across both buccal mucosa sites, underscoring the effectiveness of clobetasol in reducing discomfort associated with OLP. Similarly, a study [35] documented a downward trend in VAS scores over six weeks of clobetasol treatment, further supporting its role in sustained symptom control. Additionally, another study [36] highlighted the rapid symptomatic relief and faster healing times achieved with clobetasol ointment in orabase-B, indicating the potential benefits of using bioadhesive vehicles for drug delivery in the oral cavity.

Topical steroids have fewer side effects than systemic ones, but their severity depends on the drug formulation. Common adverse effects include candidiasis, mucosal thinning, and application discomfort [37]. Few studies [19,38] reported a brief burning sensation, local atrophy, fragility, telangiectasia, and acute candidiasis with clobetasol. Long-term use due to the chronic nature of OLP can cause these effects. The current study found no adverse effects from clobetasol or betamethasone. This was in accordance with other studies [20,23,35] who also did not report any side-effects associated with the local use of betamethasone in the treatment of OLP.

Limitations of the present study

The sample size was relatively small. Being a single-centre trial, the results may not be fully generalizable to broader populations, and influences of confounding variables should be considered. Both topical formulations were laboratory-prepared, and differences from commercially available products, particularly in excipients such as alcohols or parabens, could affect mucosal tolerability. The short follow-up duration limits long-term safety assessment. Future studies with larger, multi-centre cohorts and standardized commercial formulations are recommended to validate and expand upon these findings.

## Conclusions

Both topical Clobetasol 0.05% orabase and Betamethasone 0.05% orabase, when combined with Clotrimazole 1%, showed comparable short-term clinical improvement in the management of symptomatic OLP. Clobetasol produced a slightly better clinical response, though the difference was not statistically significant. No adverse effects were observed during the study period. Larger multi-centre studies with longer observation periods and standardized commercial formulations are recommended to validate these results.
